# Draft Genome Sequence of *Brucella melitensis* Strain ADMAS-G1, Isolated from Placental Fluids of an Aborted Goat

**DOI:** 10.1128/genomeA.00809-13

**Published:** 2013-10-10

**Authors:** Rajeswari Shome, Natesan Krithiga, Revanasiddappa Biradar Muttannagouda, Belamaranahalli Muniveerappa Veeregowda, Sahay Swati, Bibek Ranjan Shome, Udayakumar Vishnu, Jagadesan Sankarasubramanian, Jayavel Sridhar, Paramasamy Gunasekaran, Habibur Rahman, Jeyaprakash Rajendhran

**Affiliations:** Project Directorate on Animal Disease Monitoring and Surveillance, Hebbal, Bangalore, Karnataka, Indiaa; Department of Genetics, School of Biological Sciences, Madurai Kamaraj University, Madurai, Tamil Nadu, Indiab

## Abstract

Here, we report the draft genome sequence and annotation of the *Brucella melitensis* strain designated ADMAS-G1, isolated from placental fluids of an aborted goat. The length of the genome is 3,284,982 bp, with a 57.3% GC content. A total of 3,325 protein-coding genes and 63 RNA genes were predicted.

## GENOME ANNOUNCEMENT

*Brucella* species are Gram-negative facultative intracellular bacteria that cause the zoonotic disease brucellosis. *Brucella* spp. infect a broad range of mammals, from dolphins and domestic animals to humans (1). Currently, based on their host preferences and phenotypical differences, there are 10 recognized species of *Brucella*. Of these, the major species in terms of disease and impact on humans are *Brucella melitensis*, *B. suis*, *B. abortus*, and *B. canis*. *B. melitensis* is the major pathogen behind human brucellosis, and this bacterium survives intracellularly by overcoming the host defense mechanisms (2). The common virulence factors of pathogenic bacteria, such as exotoxins, secreted proteases, pili or fimbriae, cytolysins, phage-encoded proteins, flagella, and virulence plasmids, are completely missing in *Brucella* spp. (3). Therefore, whole-genome sequencing and comparative genome analysis of *B. melitensis* strains may reveal their virulence factors and pathogenic mechanisms. Hitherto, no genome sequences have been available for Indian *Brucella* isolates. Here, we present the draft genome sequence of *B. melitensis* ADMAS-G1 and its annotation.

We isolated a *B. melitensis* strain designated ADMAS-G1 from the placental fluids of an aborted goat. The 16S rRNA sequence of this strain showed 100% similarity with all *Brucella* spp. Therefore, multilocus sequence analysis (MLSA) with 9 loci was used for species identification as described previously (4). The genomic DNA of *B. melitensis* ADMAS-G1 was isolated using a DNeasy kit (Qiagen, Hilden, Germany), and the genome was sequenced using an Ion Torrent personal genome machine (Life Technologies, Carlsbad, CA). In total, 1,748,179 reads with an average read length of 157 bp were obtained, which yielded 275.68 Mb of total sequenced bases with 84.04-fold coverage. The *de novo* assembly was performed using MIRA (mimicking intelligent read assembly) version 3.4.1 (5), which yielded 19 contigs. The largest contig was 1,154,677 bp long. The draft genome sequence of *B. melitensis* ADMAS-G1 was 3,284,982 bp long, with a 57.3% GC content. The genome sequence was annotated using the RAST (Rapid Annotations using Subsystems Technology) server (6) and the NCBI Prokaryotic Genomes Annotation Pipeline (http://www.ncbi.nlm.nih.gov/genome/annotation_prok/process/). A total of 3,388 genes were predicted, of which 3,325 are protein-coding genes. Overall, 2,610 of the protein-coding genes were annotated with putative functions and 715 genes were annotated as hypothetical proteins. RAST predicted 63 RNA genes, of which 57 were tRNA and 6 were rRNA genes.

A total of 58 genes were predicted to be involved in the pathways responsible for virulence mechanisms, such as the *virB* type III, IV, and V secretory pathways and the Sec-independent protein secretion pathway component TatC, etc. In addition, 44 defense mechanism genes, which include the genes responsible for the ABC-type multidrug transport system, multidrug resistance efflux pump, and restriction endonuclease, etc., were also identified. Like other *Brucella* spp., this strain contains genes responsible for flagellum formation. But it lacks genes responsible for the chemotactic system that is necessary for assembling a functional flagellum.

### Nucleotide sequence accession numbers.

This whole-genome shotgun project has been deposited at DDBJ/EMBL/GenBank under the accession number AUTT00000000. The version described in this paper is version AUTT01000000.
